# Post-stroke Anxiety Analysis *via* Machine Learning Methods

**DOI:** 10.3389/fnagi.2021.657937

**Published:** 2021-06-25

**Authors:** Jirui Wang, Defeng Zhao, Meiqing Lin, Xinyu Huang, Xiuli Shang

**Affiliations:** ^1^Department of Neurology, The First Affiliated Hospital, China Medical University, Shenyang, China; ^2^The First Clinical Department, China Medical University, Shenyang, China; ^3^Software College, Northeastern University, Shenyang, China

**Keywords:** post-stroke anxiety, acute ischemic stroke, machine learning, random forest, risk factors analysis

## Abstract

Post-stroke anxiety (PSA) has caused wide public concern in recent years, and the study on risk factors analysis and prediction is still an open issue. With the deepening of the research, machine learning has been widely applied to various scenarios and make great achievements increasingly, which brings new approaches to this field. In this paper, 395 patients with acute ischemic stroke are collected and evaluated by anxiety scales (i.e., HADS-A, HAMA, and SAS), hence the patients are divided into anxiety group and non-anxiety group. Afterward, the results of demographic data and general laboratory examination between the two groups are compared to identify the risk factors with statistical differences accordingly. Then the factors with statistical differences are incorporated into a multivariate logistic regression to obtain risk factors and protective factors of PSA. Statistical analysis shows great differences in gender, age, serious stroke, hypertension, diabetes mellitus, drinking, and HDL-C level between PSA group and non-anxiety group with HADS-A and HAMA evaluation. Meanwhile, as evaluated by SAS scale, gender, serious stroke, hypertension, diabetes mellitus, drinking, and HDL-C level differ in the PSA group and the non-anxiety group. Multivariate logistic regression analysis of HADS-A, HAMA, and SAS scales suggest that hypertension, diabetes mellitus, drinking, high NIHSS score, and low serum HDL-C level are related to PSA. In other words, gender, age, disability, hypertension, diabetes mellitus, HDL-C, and drinking are closely related to anxiety during the acute stage of ischemic stroke. Hypertension, diabetes mellitus, drinking, and disability increased the risk of PSA, and higher serum HDL-C level decreased the risk of PSA. Several machine learning methods are employed to predict PSA according to HADS-A, HAMA, and SAS scores, respectively. The experimental results indicate that random forest outperforms the competitive methods in PSA prediction, which contributes to early intervention for clinical treatment.

## 1. Introduction

Stroke is a medical condition in which poor blood flow to the brain results in cell death, associated with high morbidity, high disability, and high mortality across the world (Wolfe, 2000). Notably, approximately 2.5 million new stroke cases annually occur in China and the mortality rate has reached 11.48% (Sun et al., 2013; Chen et al., 2017). Mood problems such as depression, apathy, and distress are commonly reported with post-stroke (Hackett et al., 2014), but anxiety in stroke patients has been relatively neglected both in clinical and research settings, in spite of its ubiquity in the general population (Remes et al., 2016). Post-stroke anxiety (PSA) refers that stroke patients extremely concern about the prognosis status, e.g., recurrence, re-working abilities, the occurrence of fall accidents, and so on (Gilworth et al., 2009). Once stroke onset, anxiety becomes common throughout the acute phase, after months, and even after years (Lincoln et al., 2013). A systematic review and meta-analysis shows that the prevalence of anxiety disorders is 29.3% post-stroke during the first year, with 36.7% in 2 weeks, 24.1% in 2 weeks to 3 months, and 23.8% in 3–12 months (Rafsten et al., 2018). Specifically, Knapp et al. (2020) collect and analyze 53 studies and report 25.5% of stroke patients developed PSA within 1 month of stroke, 23.6% in 1 and 5 months, and 21.5% in 6 months to 1 year. A plethora of studies indicate that PSA significantly influences the living quality (Lincoln et al., 2013), which is associated with the delaying recovery of neurological function (Chun et al., 2018), and the interventions on anxiety disorders have a positive impact on the incidence of both coronary artery disease and stroke (Pérez-Piñar et al., 2017).

Given the significant impact of PSA on patient outcomes, great emphasis has been placed on risk reduction and early detection. However, the pathophysiology of PSA is still unknown and the relevant risk factors are controversial. A systematic review on 18 observational studies with 8,130 patients suggests that pre-stroke depression, stroke severity, early anxiety, and dementia (or cognitive) impairment following stroke are the main predictors of PSA, while the lack of methodological and statistical rigorously affects the validity of predictive models, which indicates future research should focus on testing predictive models on both internal and external samples to ultimately inform future clinical practice (Menlove et al., 2015). Accurate individual patient risk prediction would allow for evaluation and intervention even earlier in the pathologic process. Notably, it is critical to identify risk factors associated with PSA and build models to predict PSA.

With the rapid development of advanced technology, artificial intelligence has been applied extensively in a variety of professions. As an important tool in artificial intelligence field, machine learning (Alpaydin, 2020) has received increasing attention in the last decades, which is widely utilized in medical image processing, autonomous driving, computer vision, and so on. Classic machine learning models such as linear models, decision trees (Kamiński et al., 2018), Bayesian classifiers (Kohavi, 1996), Support Vector Machines (SVM) (Cortes and Vapnik, 1995), neural networks (Müller et al., 2012), Stochastic Gradient Descent (denoted by SGD Classifier) (Zhang, 2004), Multilayer Perceptron (denoted by MLP) (Rumelhart et al., 1986), and random forests (Breiman, 2001) have exhibited certain specific usage, i.e., there are no methods suitable for solving problems at any real-life scenarios. Stochastic gradient descent is an iterative method for optimizing an objective function with suitable smoothness properties, which can be regarded as a stochastic approximation of gradient descent optimization (Saad, 1998). A multilayer perceptron is a class of feedforward artificial neural networks, which consists of at least three layers of nodes: an input layer, a hidden layer, and an output layer. MLP utilizes a supervised learning technique called backpropagation for training, which can distinguish data that is not linearly separable (Hastie et al., 2009). An SVM maps training examples to points in space so as to maximize the width of the gap between the two categories. New examples are then mapped into that same space and predicted to belong to a category based on which side of the gap they fall (Joachims, 1998). Random forest (RF), proposed by Breiman (2001), consists of a set of decision trees, each of which is a decision support tool that uses a tree-like model of decisions and their possible consequences, including chance event outcomes, resource costs, and utility (Kamiński et al., 2018). Random Forest can be used in the prediction of incident delirium (Corradi et al., 2018), malignancy of pulmonary nodules (Mei et al., 2018), survival from large echocardiography, electronic health record datasets (Samad et al., 2019), and so on. The basic thought is to determine the input sample by random sampling, and the sample data obtained will be handed over to each decision tree for judgment, thereby all the results will be voted, and the result with the most votes will be used as the output. Hence, the random forest also has ensemble learning, which can improve the accuracy of the predictive model.

Inspired by such new methods, this study plans to develop proper PSA prediction models using machine learning methods. To the best of our knowledge, this is the first study to apply machine learning to predicting anxiety for post-stroke patients. This work can identify anxiety patients after stroke at an early stage, thus benefits guiding appropriate prevention and treatments to avoid leading to severe outcomes.

The main contributions of this paper are listed as follows:

1. The main factors of PSA are analyzed in detail by traditional statistical methods between patients with/without PSA, and then all the factors with statistical difference are put into a multivariable logistic regression analysis to study in-depth.
2. Different anxiety test scales (i.e., HADS-A, HAMA, and SAS) are taken into consideration to evaluate the degree of PSA.
3. Classic machine learning methods such as decision tree and random forest are employed as predictive models to estimate PSA, and random forest outperforms the competitive approaches.

The rest of this paper is organized as follows. Section 2 introduces the material and methods for clinical data collection. Section 3 gives data analysis and experimental environment for machine learning methods. Section 4 exhibits experimental results of statistical analysis and PSA prediction comparison *via* different machine learning methods. Section 5 exhibits the discussion on the obtained results. Section 6 summarizes the whole paper and provides concluding remarks.

## 2. Materials and Methods

### 2.1. Patient Eligibility

The research protocol was accepted by the Regional Medical Scientific Research Ethics Committee of the First Affiliated Hospital of China Medical University (IRB no. 2020368). Written informed consent was obtained from all patients after a complete description of all procedures of the study provided.

From August 2017 to September 2020, 516 patients with ischemic stroke who were consecutively admitted to the stroke unit of the Department of Neurology at The First Hospital of China Medical University in China were recruited. The inclusion criteria are as follows: (1) First-ever stroke with computed tomography or magnetic resonance imaging (MRI) scan upon admission and confirmed acute cerebral infarction within 7 days after stroke onset, which meets the diagnostic criteria of 2018 Chinese Guidelines for the Diagnosis and Treatment of acute ischemic stroke (Chinese Medical Association et al., 2018); (2) age 18 years or older; (3) stable temperature, pulse, respiration, and blood pressure; and (4) signed informed consent.

Exclusion criteria are as follows: (1) Stroke-like manifestation due to definite intracranial non-vascular factors (such as primary or metastatic tumors); (2) Concurrent diagnosis of terminal illness, dementia, depression, Parkinson's disease, or motor neuron disease, all of which have been shown to cause anxiety; (3) inability to complete the scale evaluation due to communication (e.g., aphasia) or cognitive disorders; (4) administered thrombolysis therapy; (5) anxiety diagnosis before stroke; (6) inability to give informed consent. The most common reasons for exclusion were cognitive impairment (*n* = 21), depression (*n* = 19), and inability to complete the scale (*n* = 18). Thirty-two patients met other exclusion criteria and 31 patients refused to participate, leaving a total of 395 subjects (response 76.6%; 119 female, 276 male) for further analysis.

### 2.2. Collection of Clinical Data

All subjects' demographic data (gender, age, marital status, occupation, weight, and height), vascular risk factors (hypertension, coronary artery disease, diabetes mellitus, and tobacco smoking) and drinking history are collected and recorded by a trained research assistant at the time of admission. Based on the ESH/ESC hypertension guidelines recommendations (Kjeldsen et al., 2016; Cuspidi et al., 2018), hypertension is defined as systolic blood pressure (BP) ≥140 mm Hg or diastolic blood pressure higher than or equal to 90 mm Hg. Diabetes mellitus (DM) accords with the World Health Organization (WHO) diagnostic criteria for type 2 diabetes mellitus (Group et al., 1985). Smoking refers to more than 1 cigarette a day and continuous smoking for more than 3 months (Patkar et al., 2003). Drinking is distinguished by a history of more than 5 years, more than 3 times a week, and each time drinking more than 36 g of alcohol (Mazzaglia et al., 2001). Stroke severity and the level of disability were assessed using the National Institutes of Health Stroke Scale (NIHSS) (Lyden, 2017). The scores ranged from 0 (no impairment) to a maximum of 42 points. The higher the score, the more severe the neurological impairment. Scores with 4 or less are usually described as minor stroke while 21 or greater are usually described as severe stroke (Harrison et al., 2013). These measures are examined within 24 h of admission. Blood samples are obtained the morning after admission, and the serum levels of low-density lipoprotein (LDL), high-density lipoprotein (HDL), total cholesterol (TC), triglyceride (TG), glucose (GLU), uric acid (UA), C-reactive protein(CRP), creatinine (Cr), red blood cell (RBC), hemoglobin (HB), platelet (PLT), D-dimer (D-D), fibrinogen (FIB), and homocysteine (HCY) are determined.

### 2.3. Assessment of Anxiety

The degree of PSA is estimated by the Hospital Anxiety and Depression Scale (HADS-A) scores (Zigmond and Snaith, 1983), Hamilton Anxiety Scale (HAMA) scores (Hamilton, 1959), and Self-Rating Anxiety Scale (SAS) scores (Zung, 1971). The Chinese versions are validated.

As for the above-mentioned scales, the HADS-A is the most commonly used rating scale for anxiety evaluation (Burton et al., 2013). Studies have shown HADS-A correlates significantly with the Stroke Specific Quality of Life (SSQOL), with scores for energy, mood, personality, social roles, family role, thinking, and work/productivity (Rafsten et al., 2018). The HADS is a classical self-assessment mood scale specifically designed for non-psychiatric hospital departments and is presented as a reliable and valid instrument for screening for anxiety and depression after stroke (Bjelland et al., 2002). The scale is frequently used for the assessment of depression and anxiety in stroke patients (Fure et al., 2006). It includes a total of 14 items each with a score of between 0 and 3. One-half of the items are related to anxiety (HADS-A) while the other half is specific for depression. Studies have found that HADS is performed well in the assessment of both symptom severity and diagnosis of anxiety at the recommended diagnostic cut-off of ≥8 (Zigmond and Snaith, 1983; Bjelland et al., 2002). The HADS has been previously validated in Nigeria (Abiodun, 1994) where the HADS-A was found to have a sensitivity in the range of 85.0–92.9% and a specificity of 86.5–90.0%.

In order to improve the predictive accuracy of machine learning models, HAMA and SAS are also employed to screen for PSA. HAMA is a 14-items rating scale that is developed to quantify the severity of anxiety symptoms. Each item is rated on a five-point scale, ranging from 0 (not present) to 4 (severe). Total scores on the HAMA range from 0 to 56 (Maier et al., 1988). Subjects with a HAMA score equal to or larger than 7 were considered to have anxiety symptoms. SAS is a norm-referenced scale that enjoys widespread usage as a screener for anxiety disorders since developed in 1971 (Dunstan and Scott, 2018). It contains 20 items, with the score of each item ranging from 1 to 4. The greater score indicates the higher degree of anxiety that involved the conversion of a total scale raw score (with a potential range of 20–80) to an index score with a potential range of 25–100. The index score is derived by dividing the sum of the values (raw scores) obtained on the 20 items by the maximum possible score of 80, converted to a decimal and multiplied by 100 (Zung, 1971). A raw score of 40 or an index score of 50 is the cut-off of the scale (Dunstan and Scott, 2020).

## 3. Data Analysis

### 3.1. Statistical Analysis

The SPSS 26.0 statistical package (SPSS Inc., Chicago, IL) is utilized for all statistical analysis. The comparison between patients with and without PSA of continuous variables is analyzed by independent *t*-test or analyses of covariance. Univariate analyses of the association between categorical variables in both groups are performed *via* chi-square tests. Descriptive data are presented as mean and standard deviations (SD) or as 95% confidence intervals (95% CIs). When a correlation P-value is less than 0.15 for a variable, this variable is analyzed by multivariate logistic regression, and odds ratios (ORs) (with 95% CIs) are calculated for the relative risk of anxiety for each group. For all analyses, probability levels reported are two-tailed, and *P* < 0.05 is considered as the statistically significant level.

### 3.2. Prediction With Machine Learning Methods

The experiments with machine learning methods are implemented in Python 3.8.3, with relevant library Scikit-learn 0.23.2. The operating system is 64 bit Windows 10, with configuration of Intel (R) Core (TM) i7-7700 CPU @ 3.60 GHz (8 CPUs), ~3.6 GHz and 16 GB ram installed.

Machine learning methods are efficient tools for prediction and classification problems in many real-life scenarios. The general process is performed with a previous treatment and then it can be utilized to predict new cases. As the patients have completed the anxiety tests, we record the scores of each patient [denoted by *Y* = (*y*_1_, *y*_2_, ..., *y*_*n*_)] as a benchmark, the prediction result of machine methods *r* of the patients in the testing set can be represented as *Pr* = (*pr*_1_, *pr*_2_, ..., *pr*_*n*_). The Euclidean Distance (Van Der Heijden et al., 2005) can be employed to measure the similarity between the prediction results and real test scores of the *n* patients in the testing set by

(1) $$\begin{array}{l} d(Pr,Y)=\sqrt{{\left(pr_{1}-y_{1}\right)}^{2}+{\left(pr_{2}-y_{2}\right)}^{2}+...+{\left(pr_{n}-y_{n}\right)}^{2}}\\ \;=\sqrt{\sum_{i=1}^{n}{\left(pr_{i}-y_{i}\right)}^{2}} \end{array}$$

The Euclidean Distance is employed as a measure to compare the performance of each machine learning method in predicting PSA, where the smaller the Euclidean Distance, the better performance of a predicting method obtains.

## 4. Results

### 4.1. Demographic and Clinical Characteristics Between Patients With and Without PSA

Note that 395 ischemic stroke patients (119 female and 276 male between 29 and 98 years of age) are taken into consideration in the analysis. Demographic and clinical characteristics between the two groups are summarized in Tables 1–3.

**Table 1:** Significant characteristics between patients with and without post-stroke anxiety (PSA) by Hospital Anxiety and Depression Scale (HADS-A) (*n* = 395).

	**A group (*n* = 131**)	**NA group (*n* = 264**)	**Statistics**	***P*-value**
Male (yes/no)	101/30	175/89	χ^2^ = 4.862	0.027*
Age, year	60.59 ± 9.87	63.16 ± 11.40	*t* = 2.203	0.028*
Occupation	23/16/19/27/20/26	32/52/39/51/48/42	χ^2^ = 6.005	0.306
Marital status	27/34/31/39	64/79/62/59	χ^2^ = 2.929	0.403
BMI score (*kg*/*m*^2^)	23.95 ± 1.64	23.89 ± 1.26	*t* = −0.442	0.659
NIHSS score	4.26 ± 3.14	3.31 ± 2.70	*t* = −3.129	0.002*
NIHSS score group	81/50	204/60	χ^2^ = 10.389	0.001*
Hypertension (yes/no)	117/14	162/102	χ^2^ = 32.973	0.000*
CHD (yes/no)	14/117	17/247	χ2 = 2.184	0.139
DM (yes/no)	63/68	85/179	χ^2^ = 9.441	0.002*
Smoking (yes/no)	45/86	75/189	χ^2^ = 1.462	0.227
Drinking (yes/no)	34/97	36/228	χ^2^ = 9.111	0.003*
Lesion location	19/23/65/9/5/10	37/45/141/14/4/23	χ^2^ = 2.796	0.731
Lesion side	64/48/19	102/113/49	χ^2^ = 3.828	0.147
LDL-C, mmol/L	3.02 ± 1.00	3.01 ± 0.97	*t* = −0.037	0.970
HDL-C, mmol/L	1.05 ± 0.28	1.12 ± 0.28	*t* = 2.235	0.026*
TC, mmol/L	4.58 ± 1.33	4.61 ± 1.25	*t* = 0.186	0.852
TG, mmol/L	1.97 ± 1.48	1.80 ± 1.47	*t* = −1.043	0.298
GLU, mmol/L	7.59 ± 3.12	7.22 ± 3.06	*t* = −1.096	0.274
UA, umol/L	332.05 ± 70.88	339.30 ± 63.51	*t* = 0.991	0.323
CRP, mg/L	3.97 ± 2.20	3.75 ± 2.24	*t* = −0.922	0.357
Cr, umol/L	78.26 ± 26.16	78.19 ± 14.63	*t* = −0.034	0.973
RBC, *10^12/*L*	5.51 ± 0.64	5.46 ± 0.52	*t* = −0.800	0.424
HB, g/L	153.27 ± 15.81	154.07 ± 13.12	*t* = 0.530	0.596
PLT, *10^9/*L*	197.42 ± 39.96	193.78 ± 35.70	*t* = −0.406	0.685
D-D, ug/ml	0.76 ± 0.34	0.74 ± 0.32	*t* = −0.885	0.377
FIB, g/L	3.96 ± 0.82	4.05 ± 0.80	*t* = −0.554	0.580
HCY, umol/L	16.25 ± 4.71	16.24 ± 4.27	*t* = −0.012	0.991

Occupation includes staff/teacher/worker/retired/farmer/others; marital status includes divorced or separated/married/widowed/others; NIHSS score groups are divided by the number of patients with NIHSS score ≤ 4/the number of patients with NIHSS score >4. Lesion location includes lobe/basal ganglia or lateral ventricles/brainstem/cerebellum/thalamus/other; lesion side includes left/right/left and right. The data with *denote P < 0.05, which is considered as the statistically significant level. A group, anxiety group; NA group, non-anxiety group; BMI, body mass index; NIHSS, National Institutes of Health Stroke Scale; CHD, coronary heart disease; DM, diabetes mellitus; LDL, low-density lipoprotein; HDL, high-density lipoprotein; TC, total cholesterol; TG, triglyceride; GLU, glucose; UA, uric acid; CRP, C-reactive protein; Cr, creatinine; RBC, red blood cell; HB, hemoglobin; PLT, platelet; D-D, D-dimer; FIB, fibrinogen; HCY, homocysteine.

**Table 2:** Significant characteristics between patients with and without post-stroke anxiety (PSA) by Hamilton Anxiety Scale (HAMA) scale (*n* = 395).

	**A group (*n* = 133**)	**NA group (*n* = 262**)	**Statistics**	***P*-value**
Male (yes/no)	104/29	172/90	χ^2^ = 6.597	0.010*
Age, year	60.68 ± 10.06	63.13 ± 11.33	*t* = 2.102	0.036*
Occupation	23/17/18/26/21/28	32/51/40/52/44/40	χ^2^ = 6.060	0.300
Marital status	26/34/32/41	65/79/61/57	χ^2^ = 4.658	0.199
BMI score (*kg*/*m*^2^)	24.02 ± 1.58	23.85 ± 1.29	*t* = −1.181	0.238
NIHSS score	4.20 ± 3.12	3.33 ± 2.71	*t* = −2.879	0.004*
NIHSS score group	84/49	201/61	χ^2^ = 8.073	0.004*
Hypertension(yes/no)	117/16	162/100	χ^2^ = 29.055	0.000*
CHD (yes/no)	12/121	19/243	χ^2^ = 0.382	0.536
DM (yes/no)	63/70	85/177	χ^2^ = 8.388	0.004*
Smoking (yes/no)	45/88	75/187	χ^2^ = 1.132	0.287
Drinking (yes/no)	34/99	36/226	χ^2^ = 8.458	0.004*
Lesion location	20/24/63/9/5/12	36/44/143/14/4/21	χ^2^ = 3.409	0.637
Lesion side	64/50/19	102/111/49	χ^2^ = 3.265	0.195
LDL-C, mmol/L	3.00 ± 1.01	3.02 ± 0.96	*t* = 0.210	0.833
HDL-C, mmol/L	1.05 ± 0.28	1.12 ± 0.28	*t* = 2.427	0.016*
TC, mmol/L	4.56 ± 1.34	4.62 ± 1.24	*t* = 0.452	0.651
TG, mmol/L	1.95 ± 1.47	1.81 ± 1.48	*t* = −0.924	0.356
GLU, mmol/L	7.55 ± 3.10	7.24 ± 3.07	*t* = −0.939	0.348
UA, umol/L	334.49 ± 69.93	338.12 ± 64.08	*t* = 0.501	0.617
CRP, mg/L	4.00 ± 2.20	3.74 ± 2.24	*t* = −1.122	0.263
Cr, umol/L	78.94 ± 25.65	77.84 ± 14.94	*t* = −0.536	0.592
RBC, × 10^12/*L*	5.54 ± 0.62	5.45 ± 0.53	*t* = −1.386	0.167
HB, g/L	153.87 ± 13.71	153.77 ± 14.25	*t* = −0.065	0.948
PLT, × 10^9/*L*	187.40 ± 37.10	187.93 ± 35.53	*t* = 0.138	0.891
D-D, ug/ml	0.75 ± 0.34	0.75 ± 0.31	*t* = −0.068	0.946
FIB, g/L	4.00 ± 0.82	4.03 ± 0.80	*t* = 0.363	0.717
HCY, umol/L	16.44 ± 4.61	16.14 ± 4.32	*t* = −0.638	0.524

Occupation includes staff/teacher/worker/retired/farmer/others; marital status includes divorced or separated/married/widowed/others; NIHSS score groups are divided by the number of patients with NIHSS score ≤ 4/the number of patients with NIHSS score >4. Lesion location includes lobe/basal ganglia or lateral ventricles/brainstem/cerebellum/thalamus/other; lesion side includes left/right/left and right. The data with *denote P < 0.05, which is considered as the statistically significant level. A group, anxiety group; NA group, non-anxiety group; BMI, body mass index; NIHSS, National Institutes of Health Stroke Scale; CHD, coronary heart disease; DM, diabetes mellitus; LDL, low-density lipoprotein; HDL, high-density lipoprotein; TC, total cholesterol; TG, triglyceride; GLU, glucose; UA, uric acid; CRP, C-reactive protein; Cr, creatinine; RBC, red blood cell; HB, hemoglobin; PLT, platelet; D-D, D-dimer; FIB, fibrinogen; HCY, homocysteine.

**Table 3:** Significant characteristics between patients with and without post-stroke anxiety (PSA) by SAS scale (*n* = 395).

	**A group (*n* = 120**)	**NA group (*n* = 275**)	**Statistics**	***P*-value**
Male (yes/no)	93/27	183/92	χ^2^ = 4.763	0.029*
Age, year	60.84 ± 10.02	62.95 ± 11.32	*t* = 1.757	0.080
Occupation	22/15/17/24/18/24	33/53/41/54/50/44	χ^2^ = 5.937	0.312
Marital status	25/28/31/36	66/85/62/62	χ^2^ = 4.295	0.231
BMI score (*kg*/*m*^2^)	23.93 ± 1.48	23.89 ± 1.36	*t* = −0.272	0.786
NIHSS score	4.14 ± 3.03	3.40 ± 2.79	*t* = −2.379	0.018*
NIHSS score group	74/46	211/64	χ^2^ = 9.431	0.002*
Hypertension(yes/no)	108/12	171/104	χ^2^ = 31.168	0.000*
CHD (yes/no)	11/109	20/255	χ^2^ = 0.414	0.520
DM (yes/no)	61/59	87/188	χ^2^ = 13.141	0.000*
Smoking (yes/no)	41/79	79/196	χ^2^ = 1.169	0.280
Drinking (yes/no)	31/89	39/236	χ^2^ = 7.778	0.005*
Lesion location	18/22/57/9/5/9	38/46/149/14/4/24	χ^2^ = 4.603	0.466
Lesion side	57/44/19	109/117/49	χ^2^ = 2.129	0.345
LDL-C, mmol/L	3.05 ± 1.01	3.00 ± 0.97	*t* = −0.512	0.609
HDL-C, mmol/L	1.04 ± 0.27	1.12 ± 0.28	*t* = 2.552	0.011*
TC, mmol/L	4.59 ± 1.33	4.60 ± 1.25	*t* = 0.079	0.937
TG, mmol/L	1.98 ± 1.53	1.80 ± 1.45	*t* = −1.100	0.272
GLU, mmol/L	7.55 ± 3.08	7.25 ± 3.08	*t* = −0.896	0.371
UA, umol/L	332.85 ± 69.22	338.66 ± 64.65	*t* = 0.804	0.422
CRP, mg/L	3.96 ± 2.20	3.77 ± 2.24	*t* = −0.766	0.444
Cr, umol/L	79.23 ± 26.62	77.77 ± 14.90	*t* = −0.697	0.486
RBC, × 10^12/*L*	5.53 ± 0.60	5.46 ± 0.55	*t* = −1.062	0.289
HB, g/L	153.82 ± 13.83	153.80 ± 14.17	*t* = −0.008	0.993
PLT, × 10^9/*L*	187.41 ± 36.33	187.90 ± 35.95	*t* = 0.124	0.901
D-D, ug/ml	0.77 ± 0.34	0.74 ± 0.31	*t* = −0.723	0.470
FIB, g/L	4.01 ± 0.80	4.03 ± 0.81	*t* = 0.156	0.876
HCY, umol/L	16.53 ± 4.69	16.12 ± 4.30	*t* = −0.847	0.397

Occupation includes staff/teacher/worker/retired/farmer/others; marital status includes divorced or separated/married/widowed/others; NIHSS score groups are divided by the number of patients with NIHSS score ≤ 4/the number of patients with NIHSS score >4. Lesion location includes lobe/basal ganglia or lateral ventricles/brainstem/cerebellum/thalamus/other; lesion side includes left/right/left and right. The data with *denote P < 0.05, which is considered as the statistically significant level. A group, anxiety group; NA group, non-anxiety group; BMI, body mass index; NIHSS, National Institutes of Health Stroke Scale; CHD, coronary heart disease; DM, diabetes mellitus; LDL, low-density lipoprotein; HDL, high-density lipoprotein; TC, total cholesterol; TG, triglyceride; GLU, glucose; UA, uric acid; CRP, C-reactive protein; Cr, creatinine; RBC, red blood cell; HB, hemoglobin; PLT, platelet; D-D, D-dimer; FIB, fibrinogen; HCY, homocysteine.

On the whole, in the PSA group, the mean age of patients is relatively younger and serum HDL-C level is lower. The proportion of male patients, serious stroke, hypertension, diabetes mellitus, and drinking were significantly higher in the PSA group than the non-anxiety group.

### 4.2. Multivariate Logistic Regression Analyses of the Risk Factors Associated With PSA

As exhibited in Table 4, gender, age, NIHSS score, hypertension, CHD, DM, drinking, and HDL-C are fed into the multivariate logistic regression model by HADS-A. As exhibited in Tables 5, 6, gender, age, NIHSS score, hypertension, DM, drinking, and HDL-C are fed into the multivariate logistic regression model by HAMA and SAS scale, respectively. Multivariate logistic regression (stepwise forward) analysis indicates that hypertension, diabetes mellitus, drinking, high NIHSS score, and low serum HDL-C level are associated with PSA, as shown in Tables 4–6.

**Table 4:** Multivariate logistic regression analyses of the risk factors associated with post-stroke anxiety (PSA) evaluated by Hospital Anxiety and Depression Scale (HADS-A) scale.

	**B**	**S.E**.	**Wald**	**OR**	**OR 95% CI**	***P*-value**
Constant	−1.882	0.583	10.473	0.152		0.001
Hypertension	1.843	0.333	30.636	6.318	3.289–12.136	0.000
DM	0.756	0.241	9.814	2.129	1.327–3.416	0.002
Drinking	1.171	0.306	14.603	3.324	1.769–5.877	0.000
HDL-C	−0.961	0.442	4.733	0.382	0.161–0.909	0.030
NIHSS score group	0.863	0.225	11.429	2.371	1.437–3.912	0.001

*NIHSS score groups are divided by the number of patients with NIHSS score ≤ 4/the number of patients with NIHSS score >4. OR, odds ratio; DM, diabetes mellitus; HDL, high-density lipoprotein; NIHSS, National Institutes of Health Stroke Scale*.

**Table 5:** Multivariate logistic regression analyses of the risk factors associated with post-stroke anxiety (PSA) evaluated by Hamilton Anxiety Scale (HAMA) scale.

	**B**	**S.E**.	**Wald**	**OR**	**OR 95% CI**	***P*-value**
Constant	−1.535	0.566	7.363	0.216		0.007
Hypertension	1.647	0.315	27.419	5.193	2.803–9.621	0.000
DM	0.689	0.237	8.423	1.991	1.251–3.170	0.004
Drinking	1.105	0.301	13.515	3.020	1.675–5.444	0.000
HDL-C	−1.034	0.437	5.592	0.356	0.151–0.838	0.018
NIHSS score group	0.744	0.251	8.752	2.104	1.285–3.444	0.003

*NIHSS score groups are divided by the number of patients with NIHSS score ≤ 4/the number of patients with NIHSS score >4. OR, odds ratio; DM, diabetes mellitus; HDL, high-density lipoprotein; NIHSS, National Institutes of Health Stroke Scale*.

**Table 6:** Multivariate logistic regression analyses of the risk factors associated with post-stroke anxiety (PSA) evaluated by SAS scale.

	**B**	**S.E**.	**Wald**	**OR**	**OR 95% CI**	***P*-value**
Constant	−1.949	0.605	10.359	0.142		0.001
Hypertension	1.888	0.353	28.530	6.605	3.304–13.205	0.000
DM	0.906	0.247	13.474	2.474	1.525–4.013	0.000
Drinking	1.145	0.311	13.552	3.141	1.708–5.777	0.000
HDL-C	−1.133	0.460	6.077	0.322	0.131–0.793	0.014
NIHSS score group	0.851	0.260	10.680	2.342	1.406–3.902	0.001

*NIHSS score groups are divided by the number of patients with NIHSS score ≤ 4/the number of patients with NIHSS score >4. OR, odds ratio; DM, diabetes mellitus; HDL, high-density lipoprotein; NIHSS, National Institutes of Health Stroke Scale*.

### 4.3. Post-stroke Anxiety *via* Machine Learning Methods

To compare the performance of classic machine learning methods in PSA prediction, we carry out *k*-fold cross-validation by splitting the dataset into *k* parts. One part is assigned as the testing set and the remaining parts are regarded as training set each time until each part has already been calculated. Therefore, the validation process needs *k*-times comparison. Suppose *k* = 10, each machine learning method is employed to predict anxiety test in the *k*-cross validation test, and the results are shown in Table 7.

**Table 7:** Averaging Euclidean distance of 10-fold cross-validation on the five machine learning methods by comparing with Hospital Anxiety and Depression Scale (HADS-A), Hamilton Anxiety Scale (HAMA), and SAS test, respectively.

	**RandomForest**	**SVM**	**DecisionTree**	**SGDClassifier**	**MLP**
HADS-A	15.7433	17.7001	74.9658	75.1603	99.0425
HAMA	18.0221	20.6518	101.3920	104.0841	123.5836
SAS	22.1108	29.4717	133.6214	133.6214	169.5637

**Figure 1:**
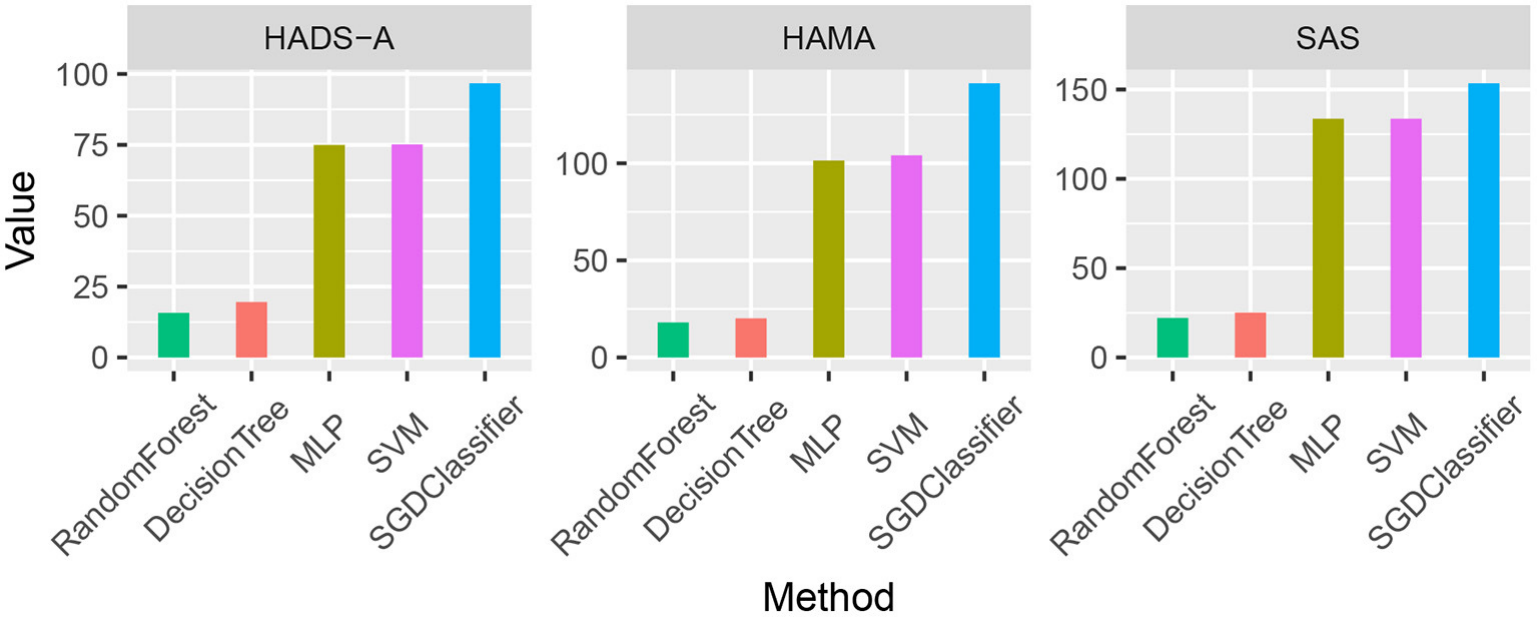
Averaging Euclidean Distance comparison of the five machine learning methods by Hospital Anxiety and Depression Scale (HADS-A), Hamilton Anxiety Scale (HAMA), and SAS tests, respectively.

As shown in Table 7, the averaging Euclidean Distance obtained by RandomForest method is 18.6254, which outperforms than methods (22.6079, 103.3264, 104.2886, and 130.7300, respectively) on HADS-A test. Likewise, the competitive methods are evaluated by HAMA and SAS tests, which also suggests the superiority of random forest. We plotted the averaging Euclidean distance of each machine learning methods as shown in Figure 1.

**Figure 2:**
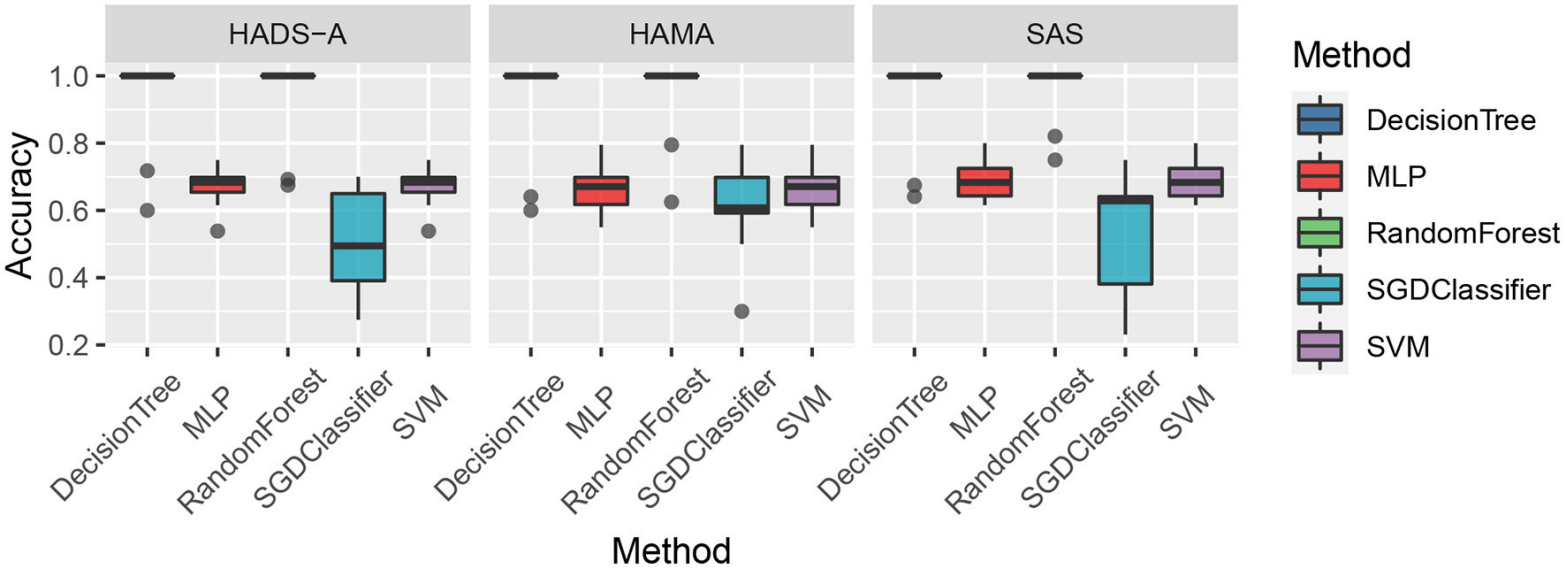
Accuracy comparison of the five machine learning methods by Hospital Anxiety and Depression Scale (HADS-A), Hamilton Anxiety Scale (HAMA), and SAS tests, respectively.

As shown in Figure 1, the random forest method has a lower averaging Euclidean distance than the competitors, which indicates its superiority in predicting PSA. To compare the predicting accuracy of the above-mentioned methods, we compared the results and the boxplot is shown in Figure 2.

**Figure 3:**
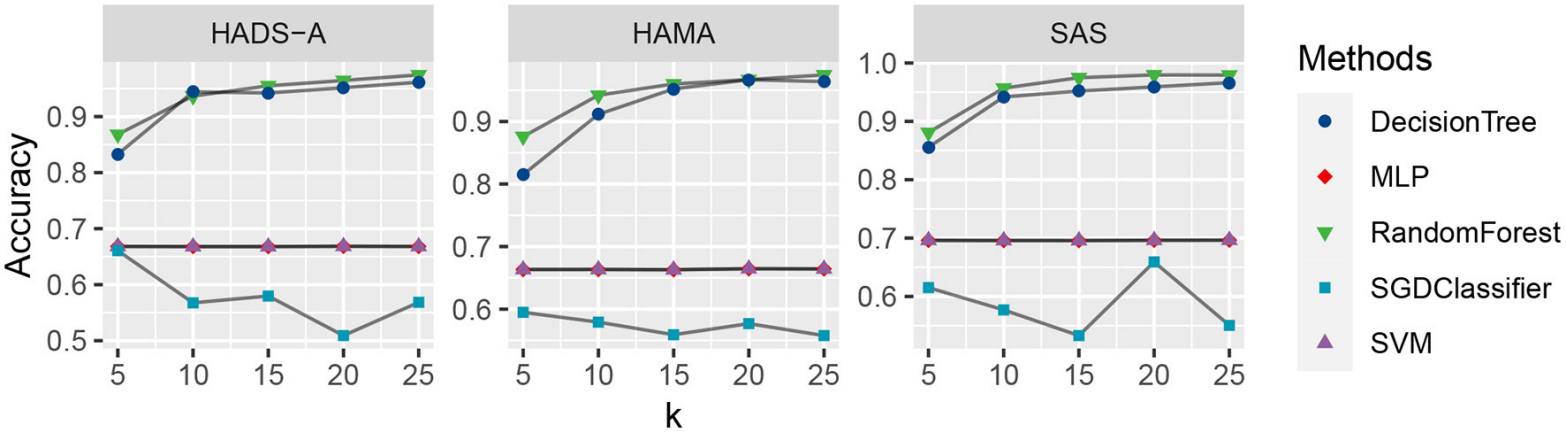
Accuracy comparison of the five machine learning methods with varying *k*, evaluated by Hospital Anxiety and Depression Scale (HADS-A), Hamilton Anxiety Scale (HAMA), and SAS tests, respectively.

As shown in Figure 2, the decision tree and random forest methods are holding higher accuracy in predicting PSA. Specifically, the abnormal values of random forest method are higher than that of decision tree, which shows the superiority of ensemble learning. To analyze the relationship between accuracy and the varying *k* in the *k*-cross-validation process, we conduct the experiments with different *k*, i.e., *k* = {5, 10, 15, 20, 25}, and plot the result as shown in Figure 3.

As shown in Figure 3, the random forest method (marked with green triangles) outperforms the competitive methods with *k* increasing in all the three anxiety scales. The DecisionTree method is second to RandomForest, and MLP (marked with red rhombus) and SVM (marked with purple triangles) are at the same level and show robustness with *k* increasing. The SGDClassifier (marked with blue square) is inferior to the other methods in general, which suggests more treatment or optimization of SGDClassifier is needed for further analysis.

## 5. Discussion

With the development of stroke relevant research, post-stroke emotional disorder has attracted more and more attention. The identification of risk factors benefits detecting PSA at an early stage and achieving timely intervention. The statistics in this study show that the frequency of PSA is 33.16% evaluated by HADS-A, 33.67% by HAMA, and 30.38% by SAS, respectively. Our results are consistent with the findings in previous studies (Burton et al., 2013; Broomfield et al., 2014; Knapp et al., 2017, 2020; Rafsten et al., 2018), i.e., 18–36.7% of patients with acute ischemic stroke experienced anxiety 0–2 weeks after stroke onset.

The averaging age of PSA patients is younger than that of patients without PSA by HADS-A and HAMA scale, but there is no statistical difference in SAS evaluation, as shown in Tables 1–3. A systematic review of observational studies revealed that older age was the most consistent factor not predictive of PSA (Menlove et al., 2015). This may result from a combination of anxiety disorders being much less common in older adults while an increasing proportion risk of stroke in older adults (McEvoy et al., 2011). Researchers also propose that younger people especially those with a history of anxiety or depression are more probably to have PSA (Chun et al., 2018). Thus, the above-mentioned different viewpoints seem to explain the results of the multivariate analysis in section 4, which shows that age is not a risk factor of PSA. This study exhibits that men are more probably to be anxious after stroke than women, as shown in single factor analysis in Tables 1, 2. Burton et al. (2013) declare that 51–64% of PSA are male patients. As shown in multivariate logistic analysis, drinking is an independent risk factor of PSA. Notably, male patients are more likely to drink, which may support Burton's viewpoint. On the contrary, Beauchamp et al. (2020) insist that PSA is more common in female stroke patients while gender is analyzed in univariable analysis, but gender is not a statistically significant factor in their or our multivariable analysis. Thus, just as other precious researchers suggested (Astrom, 1996; Schultz et al., 1997; Leppävuori et al., 2003; Shuibin, 2006; Barker-Collo, 2007; Carod-Artal et al., 2009; Sagen et al., 2010), the relation between age and PSA is not sure and caution should be observed when making conclusions on the association of gender and PSA. Besides, the NIHSS scores of PSA patients are more prone to be higher than that of patients without anxiety in our study, suggesting that the severity of stroke is a risk factor for PSA, which is consistent with previous results (Menlove et al., 2015). It is clear that social isolation, loneliness, and single status are linked to higher rates of cardiovascular disease and stroke mortality and morbidity (Tillmann et al., 2017; Hakulinen et al., 2018), and the association between PSA and non-married status has also been revealed (Beauchamp et al., 2020). As for marital status in our study, different from our subjective clinical experience or above studies, there are no significant differences between different marital statuses. Since a large number of patients are reluctant to reveal their concrete marital status and we grouped these patients into “others,” this result may generate false-negative data. The relationship between PSA and affected brain regions is controversial. We find no association between lesion location or lesion side and PSA, which is the same with Chun et al. (2018), and a meta-analysis about PSA (Burton et al., 2013) summarized that no association was observed between PSA and lesion location in five of six studies (Astrom, 1996; Ghika-Schmid et al., 1999; Leppävuori et al., 2003; Fure et al., 2006; Barker-Collo, 2007). On the contrary, Tang et al. (2012) reported that PSA patients were more likely to have right frontal acute infarcts compared with non-PSA group. Differences in the above results may due to the small sample size, lack of detailed assessment of lesion locations and the diversity between CT and MRI scans to estimate lesion locations.

This study also revealed that patients with hypertension or diabetes mellitus are more prone to have PSA. A cross-sectional study also found that chronic physical diseases is an identified factor significantly associated with post-stroke mental health (Almhdawi et al., 2020). Interestingly, our study found that the level of HDL-C is independent protective factors for PSA. Although relevant research is rare and the mechanism is unclear, it has been commonly acknowledged that a higher level of HDL-C is a protective factor for stroke, and the protective mechanism of HDL-C for PSA may be the same as that for stroke.

Machine learning methods are commonly applied in modern medical research, such as image processing, computer-aided diagnosis, and so on. It is really a challenging task to predict PSA with limited clinical data and it is an open issue. To the best of our knowledge, there are bare research about predicting PSA *via* machine learning methods. In our study, machine learning methods are employed to predict PSA, and anxiety scales are utilized as evaluation benchmarks. As shown in Figure 1, random forest methods have the averaging Euclidean Distance of 18.6254 in HADS-A, HAMA, and SAS scales, being superior to that of DecisionTree, SVM, SGDClassifier, and MLP (i.e., 22.6079, 103.3264, 104.2886, and 130.7300, respectively), which is consistent with the findings in Tripathi et al. (2019). As plotted in Figure 2, decision tree and random forest methods show higher accuracy than the other three methods. In the *k*-fold cross-validation process, we set *k* = 10 and collect all the accuracy results, the abnormal values of decision tree (i.e., 0.6410 and 0.7250 in HADS-A, 0.5500 and 0.6410 in HAMA, 0.5897 and 0.7250 in SAS) and random forest (i.e., 0.6750 and 0.6923 in HADS-A, 0.6250 and 0.7949 in HAMA, 0.7500 and 0.8205 in SAS) are obvious, which indicate the optimization of training process is expected in future works. In a word, with the aid of ensemble learning, random forest can be applied in PSA prediction. Figure 3 shows the relationship between *k* and accuracy from different anxiety scales. With the increasing of *k*, the predictive accuracy of machine learning methods improves gradually. The RandomForest and DecisionTree methods are at the same level, outperform the other three methods. RandomForest methods show superiority to DecisionTree, with a slender advantage in general, which further indicates the capability of RandomForest methods in predicting PSA.

The limitations of this study are listed as follows. In section 4, the risk factors of PSA are analyzed merely by tables, other variables potentially associated with PSA are not under consideration. For example, a review (Popa-Wagner et al., 2020) summarized plenty of articles pointed that lifestyle (such as high sugar diets, high fat diets or calorie restriction) can influence the onset, severity, and duration of the stroke, so it will be interesting and meaningful to study the relationship between lifestyle and PSA. Slevin et al. (2015) demonstrated that mCRP may be responsible for promoting dementia after ischemia stroke by sufficient *in vitro* experiments, murine models, and detailed histological studies, emphasizing the influence of inflammation on stroke and suggesting that the relationship between systemic inflammation and PSA should be further studied. Besides, since previous studies (Burton et al., 2013; Menlove et al., 2015) have reported significant associations between PSA and Pre-stroke depression, aphasia, dementia, or cognitive impairment, we excluded these patients in our study and this selection bias may limit the generalizability of the findings. Thus, all the above would be further studied in the forthcoming research.

## 6. Conclusion

Anxiety after stroke is common and disabling (Chun et al., 2018), which may lead to severe effects and bring great troubles to patients. In this paper, we carry out a series of experiments to analyze the risk factors and employ machine learning methods to predict PSA. The experimental results suggest that hypertension, diabetes mellitus, drinking, disability, and low serum HDL-C levels are closely related to anxiety in acute ischemic stroke, and random forest can be applied in PSA prediction. These results not only provide insight into the possible factors related to PSA but also benefit predicting anxiety of acute ischemic stroke patients, providing a theoretical basis for the treatment of PSA. It is of great significance in lowering costs of care by shortening the course of treatment or reducing the possibility of anxiety with the aid of the findings in this work, and we hope it will shed light on more forthcoming researchers to further explore the uncharted part of this promising field.

## Data Availability Statement

The original contributions presented in the study are included in the article, further inquiries can be directed to the corresponding author.

## Ethics Statement

The studies involving human participants were reviewed and approved by the Regional Medical Scientific Research Ethics Committee of the First Affiliated Hospital of China Medical University (IRB no.2020368). The patients/participants provided their written informed consent to participate in this study.

## Author Contributions

JW wrote the original draft. DZ designed the post-stroke anxiety evaluation for all the collected cases. ML revised the manuscript. XH conducted the machine learning experiments and plotted the figures. XS checked the manuscript and made final modifications. All authors contributed to the article and approved the submitted version.

## Conflict of Interest

The authors declare that the research was conducted in the absence of any commercial or financial relationships that could be construed as a potential conflict of interest.
